# Myocardial injury in diabetic patients with multivessel coronary artery disease after revascularization interventions

**DOI:** 10.1186/s13098-017-0292-3

**Published:** 2017-11-21

**Authors:** Paulo Cury Rezende, Whady Hueb, Rosa Maria Rahmi, Thiago Luis Scudeler, Diogo Freitas Cardoso de Azevedo, Cibele Larrosa Garzillo, Carlos Alexandre Wainrober Segre, Jose Antonio Franchini Ramires, Roberto Kalil Filho

**Affiliations:** Heart Institute (InCor) of the University of São Paulo, Avenida Dr. Eneas de Carvalho Aguiar, 44, AB sala 114, Cerqueira César, São Paulo, SP CEP 05403-900 Brazil

**Keywords:** Type 2 diabetes mellitus, Coronary artery disease, Percutaneous coronary intervention, Coronary artery bypass, Myocardial infarction

## Abstract

**Background:**

Diabetic patients may be more susceptible to myocardial injury after coronary interventions. Thus, the aim of this study was to assess the release of cardiac biomarkers, CK-MB and troponin, and the findings of new late gadolinium enhancement (LGE) on cardiac magnetic resonance (CMR) in patients with type 2 diabetes mellitus after elective revascularization procedures for multivessel coronary artery disease (CAD).

**Methods:**

Patients with multivessel CAD and preserved systolic ventricular function underwent either elective percutaneous coronary intervention (PCI), off-pump or on-pump bypass surgery (CABG). Troponin and CK-MB were systematically collected at baseline, 6, 12, 24, 36, 48 and 72 h after the procedures. CMR with LGE was performed before and after the interventions. Patients were stratified according to diabetes status at study entry. Biomarkers and CMR results were compared between diabetic and nondiabetics patients. Analyses of correlation were also performed among glycemic and glycated hemoglobin (A1c) levels and troponin and CK-MB peak levels. Patients were also stratified into tertiles of fasting glycemia and A1c levels and were compared in terms of periprocedural myocardial infarction (PMI) on CMR.

**Results:**

Ninety (44.5%) of the 202 patients had diabetes mellitus at study entry. After interventions, median peak troponin was 2.18 (0.47, 5.14) and 2.24 (0.69, 5.42) ng/mL (*P* = 0.81), and median peak CK-MB was 14.1 (6.8, 31.7) and 14.0 (4.2, 29.8) ng/mL (*P* = 0.43), in diabetic and nondiabetic patients, respectively. The release of troponin and CK-MB over time was statistically similar in both groups and in the three treatments, besides PCI. New LGE on CMR indicated that new myocardial fibrosis was present in 18.9 and 17.3% (*P* = 0.91), and myocardial edema in 15.5 and 22.9% (*P* = 0.39) in diabetic and nondiabetic patients, respectively. The incidence of PMI in the glycemia tertiles was 17.9% versus 19.3% versus 18.7% (*P* = 0.98), and in the A1c tertiles was 19.1% versus 13.3% versus 22.2% (*P* = 0.88).

**Conclusions:**

In this study, diabetes mellitus did not add risk of myocardial injury after revascularization interventions in patients with multivessel coronary artery disease.

*Trial Registration* Name of Registry: Evaluation of cardiac biomarker elevation after percutaneous coronary intervention or coronary artery bypass graft; URL: http://www.controlled-trials.com.ISRCTN09454308

## Background

Myocardial revascularization interventions are routinely indicated during the course of the treatment of patients with chronic coronary artery disease (CAD). The most important benefit observed with these interventions is the improvement in angina and exercise capacity in patients with limiting symptoms [1, 2].

However, during these interventions, myocardial damage may occur, and this may also influence outcomes during follow-up [3]. Periprocedural myocardial injury (PMI) has been extensively studied but is still not completely understood. The diagnosis is still challenging, and the appropriate treatment is yet to be defined. Especially with the emergence of highly sensitive markers of myocardial necrosis, the diagnosis of myocardial injury and infarction after coronary interventions has become even more debatable [4, 5].

Many known and unknown factors may influence myocardial injury following interventions. Although this is still controversial, diabetes mellitus may change myocardial functions and potentially make the myocardium more susceptible to ischemic injuries [6].

Experimental studies that addressed this question have shown contradictory results about the ischemic myocardial sensitivity of diabetic animal models. While some studies have demonstrated higher cardiac damage after ischemic insults [7, 8], others have shown no differences [9] or even a protective role of diabetes [10–12].

Additionally, clinical studies in the scenario of acute coronary syndromes have shown indirect evidence of a higher susceptibility of diabetic patients to such things as greater risk for the development of heart failure [13] and higher morbidity and mortality rates [14]. However, uncertainties still remain about whether these are caused by a higher sensitivity to myocardial ischemia due to diabetes mellitus, or if these are related to other reasons such as more diffuse and aggressive coronary disease or due to the microvascular complications potentially caused by diabetes. Moreover, direct evidence from human studies about myocardial responses to ischemic insults in diabetic populations is still inconclusive and scarce.

In this context, the medicine, angioplasty or surgery study V is a prospective trial that assessed myocardial injury after myocardial revascularization interventions in patients with chronic multivessel CAD who underwent percutaneous coronary intervention (PCI), on-pump, or off-pump coronary artery bypass surgery (CABG) [15]. Cardiac biomarkers, troponin, and CK-MB were systematically collected before and after procedures to assess myocardial injury by the perspective of high-sensitive biomarkers released after interventions. Patients also underwent cardiac magnetic resonances (CMR) with late gadolinium enhancement (LGE) before and after procedures to assess new areas of myocardial infarction by the perspective of a high-sensitive imaging study. In this post hoc analysis, myocardial injury was analyzed in patients with type 2 diabetes mellitus and compared with patients without diabetes.

## Methods

The medicine, angioplasty, or surgery study V (MASS-V) is a prospective nonrandomized trial that aimed at studying PMI after PCI, on-pump, or off-pump CABG [15]. Patients with angiographically documented multivessel coronary stenosis of more than 70% by visual assessment and myocardial ischemia due to angina symptoms or positive stress tests were included. Only patients with stable symptoms and preserved systolic ventricular function were included. They were excluded if they have experienced a recent acute coronary syndrome or other thromboembolic phenomena in the last 3 months, systemic inflammatory disease, or kidney dysfunction (creatinine above 2.0 mg/mL).

### Cardiac biomarkers

Blood samples were collected for troponin I (TnI) and CK-MB measurement before procedures and 6, 12, 24, 36, and 48 h after PCI. After on-pump or off-pump CABG, these biomarkers were measured before and 6, 12, 24, 36, 48, and 72 h after surgery.

All samples were centrifuged at 3000 rpm for 20 min and analyzed within 2 h after collection. Analyses of TnI and CK-MB were performed using an ADVIA Centaur immunoassay analyser (Siemens Health Care Diagnostics, Tarrytown, NY). According to the manufacturer, the lower limit of detection of TnI using the high-sensitivity Ultra kit is 0.006 ng/mL, and the 99th percentile upper reference limit (URL) is 0.04 ng/mL. The assay precision represented by the percentage coefficient of variation is 10% at 0.03 ng/mL. The detection limit of the CK-MB mass kit is 0.18 ng/mL. Cutoff values at the 99th percentile are 3.8 ng/mL for women and 4.4 ng/mL for men. The coefficients of variations for CK-MB mass as specified by the manufacturer are 3.91% at 3.55 ng/mL and 3.67% at 80.16 ng/mL. These measurements are in accordance with the recommendations of the Study Group on Biomarkers in Cardiology of the European Society of Cardiology Working Group on Acute Cardiac Care [16].

### Cardiac magnetic resonance

Every patient underwent a CMR before and after interventions during the hospitalization period. They were studied in a 1.5-T Achieva Magnetic Resonance scanner (Philips Healthcare, Andover, MA). Steady-state free-procession cine images were acquired in two long-axis (two and four chambers) views and 8–10 short-axis views of the left ventricle. Contrast-enhanced images were acquired in long- and short-axis planes identical to the cine images. Typical voxel size was 1.6, 2.1, and 8 mm, with a reconstruction matrix of 528 and a reconstructed voxel size of 0.6 mm. Myocardial infarction was defined as the identification of hyperenhancement in the myocardium on CMR. Infarcted regions exhibit this phenomenon, which might be due to an increased volume of distribution of the contrast agent, because of rupture of myocyte membranes and slow contrast washout.

The areas of late gadolinium hyperenhancement were measured by two experienced and independent observers blinded to the intervention technique and biomarker data. When measurements differed, a third observer performed a review, and a consensus was obtained. Hyperenhanced pixels were defined as those with image intensities exceeding two standard deviations greater than the mean of image intensities in a remote myocardial region in the same image. Preintervention and postintervention scans were read side by side in the three intervention groups.

### Definition of type 2 diabetes mellitus

Patients were considered to have diabetes if, at baseline, they were using insulin and/or oral hypoglycemic agents, of if they had the classical criteria for type 2 diabetes mellitus as stated by the American Diabetes Association [17] (two fasting glucose measures ≥ 126 mg/dL, glycated hemoglobin [A1c] ≥ 6.5%, random glucose ≥ 200 mg/dL, or 2-h plasma glucose ≥ 200 mg/dL during an oral glucose tolerance test).

### Statistical analysis

Categorical data are presented as percentages and continuous data as means ± standard deviations, or as medians and interquartile ranges, as appropriate. Categorical data were compared using the Chi square test or Fisher’s exact test. Continuous variables were tested regarding their distribution with the Shapiro–Wilk test. Those normally distributed were compared using the unpaired *t* test and if not normally distributed by Wilcoxon rank-sum test. The comparison of troponin and CK-MB over time between diabetic and nondiabetic patients was also performed using the Wilcoxon rank-sum test. Analyses of correlations were performed among glycemia and A1c levels and troponin and CK-MB, and nonparametric Spearman’s correlation coefficients were calculated with their 95% confidence intervals. All tests were 2-sided and a *P* value < 0.05 was considered statistically significant. Graphs were created in Graphpad Prism 7.0a, 2016, and all analyses were performed using R software (2016, R Foundation for Statistical Computing, Vienna, Austria, URL https://www.R-project.org/).

## Results

Between May 2012 and March 2014, 326 patients with multivessel CAD were analyzed for study enrollment. From these, 219 were included in this prospective, single-center, nonrandomized trial and were assigned to PCI, on-pump or off-pump CABG. Seventeen patients were excluded after the procedures (11 due to claustrophobia during CMR, four due to cerebrovascular accidents, and two due to septicemia). Thus, 202 patients were analyzed in this study. Of these, 66 were assigned to PCI, 67 to off-pump, and 69 to on-pump CABG. Ninety patients (44.5%) had diabetes mellitus at study entry. The baseline characteristics of diabetic and nondiabetic patients are presented in Table 1. Both groups were statistically similar regarding main demographic characteristics, despite glycemic levels. Table 2 shows the SYNTAX scores of patients with and without diabetes, stratified by treatment groups.

**Table 1:** Baseline demographic variables of diabetic and nondiabetic patients in the MASS V trial

	Diabetic patients n = 90	Nondiabetic patients n = 112	*P*
Male (%)	68.9	66.1	0.78
Age (year)^a^	62.5 (55.0, 67.75)	62.0 (56.0, 68.0)	0.96
Hypertension (%)	86.7	83.0	0.61
Prior MI (%)	31.1	33.0	0.89
Angina (%)	89.9	89.3	1.0
3-Vessel CAD (%)	72.2	62.5	0.28
LAD disease (%)	88.9	90.2	0.95
LM disease (%)	20.0	25.9	0.41
SYNTAX score^a^	21.0 (16.0, 26.9)	19.0 (14.0, 25.0)	0.27
EF^a^	0.67 (0.61, 0.75)	0.67 (0.61, 0.72)	0.56
Cholesterol^a^	165.0 (134.0, 189.0)	168.0 (147.5, 197.0)	0.18
LDL-Cholest^a^	93.0 (72.0, 117.0)	98.0 (78.0, 120.8)	0.17
HDL-Cholest^a^	37.0 (30.0, 42.0)	35.0 (29.3, 46.0)	0.74
Triglycerides^a^	134.0 (91.0, 196.0)	128.0 (96.5, 167.5)	0.95
Glucose^a^	146.5 (116.2, 194.5)	100.0 (94.0, 112.0)	< 0.001
A1c^a^	6.9 (6.05, 8.50)	–	–
Creatinine^a^	1.00 (0.88, 1.22)	0.98 (0.85, 1.20)	0.65

*MI* myocardial infarction, *CAD* coronary artery disease, *LAD* left anterior descending coronary artery, *LM* left main coronary artery, *EF* ejection fraction, *LDL* low-density lipoprotein, *HDL* high-density lipoprotein, *A1c* glycated hemoglobin

^a^Presented as median (25th, 75th percentile)

**Table 2:** SYNTAX scores in patients with and without diabetes, in the three treatment groups

	On-pump CABG	Off-pump CABG	PCI
Diabetic patients	24.0 (18.0, 30.0)	21.0 (15.5, 25.5)	18.0 (11.0, 22.7)
Nondiabetic patients	25.0 (18.7, 31.4)	19.0 (14.7, 23.0)	15.5 (11.0, 20.7)

Data is presented as median (25th, 75th percentile)

*CABG* coronary artery bypass graft surgery, *PCI* percutaneous coronary intervention

### Biomarkers and CMR assessment

The study protocol assessed troponin and CK-MB at baseline, 6, 12, 24, 36, and 48 h after PCI and in the same periods up to 72 h after on-pump and off-pump CABG in 202 CAD patients. A total of 2658 biomarker samples were analyzed in this study.

After PCI, we analyzed 386 troponin samples until 48 h (97.5% of all 396 possible measurements). Regarding CK-MB, we analyzed 383 samples (96.7% of all 396 possible samples).

After on-pump and off-pump CABG, we analyzed 942 troponin samples until 72 h (98.9% of all 952 possible measurements). Regarding CK-MB, we analyzed 947 samples (99.5% of all 952 possible samples).

All 202 patients underwent 2 CMRs with LGE, and thus 404 CMRs were analyzed regarding new areas of myocardial fibrosis and myocardial edema after coronary interventions.

### Troponin results

The group of diabetic patients had a mean peak troponin of 5.38 ng/mL with a standard deviation of 9.49 ng/mL, and a median and interquartile range (IQR) of 2.18 ng/mL (0.47, 5.14). The group of nondiabetic patients had a mean peak troponin of 5.91 ng/mL with a standard deviation of 9.88 ng/mL, and median and IQR of 2.24 ng/mL (0.69, 5.42). The total area under the curve was also statistically similar between both groups (*P* = 0.97).

**Fig. 1:**
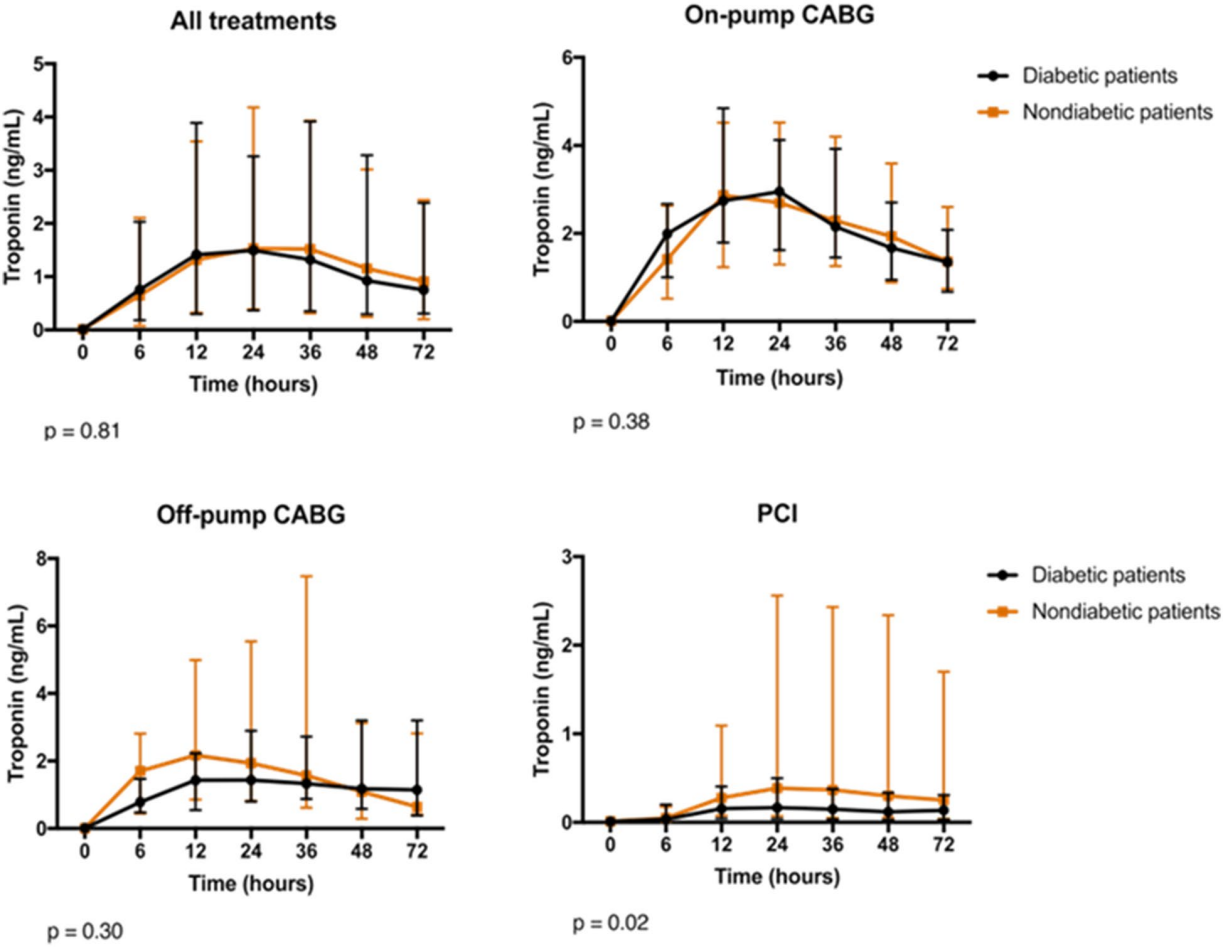
Troponin results at baseline and after 6, 12, 24, 36, 48, and 72 h after coronary interventions in diabetic and nondiabetic patients. Data are presented as medians and interquartile ranges. *P* values are provided by the comparison of both groups regarding all time points

We also compared troponin values at baseline and during each time point (6, 12, 24, 36, 48, and 72 h) between diabetic and nondiabetic patients and separately for each cardiac procedure (Fig. 1).

### CK-MB results

The group of diabetic patients had a mean peak CK-MB of 27.8 ng/mL with a standard deviation of 37.3 ng/mL, and a median and IQR of 14.1 ng/mL (6.8, 31.7). The group of nondiabetic patients had a mean peak CK-MB of 25.2 ng/mL with a standard deviation of 34.1 ng/mL, and median and IQR of 14.0 ng/mL (4.2, 29.8), *P* = 0.43. The total area under the curve was also statistically similar in both groups (*P* = 0.56).

**Fig. 2:**
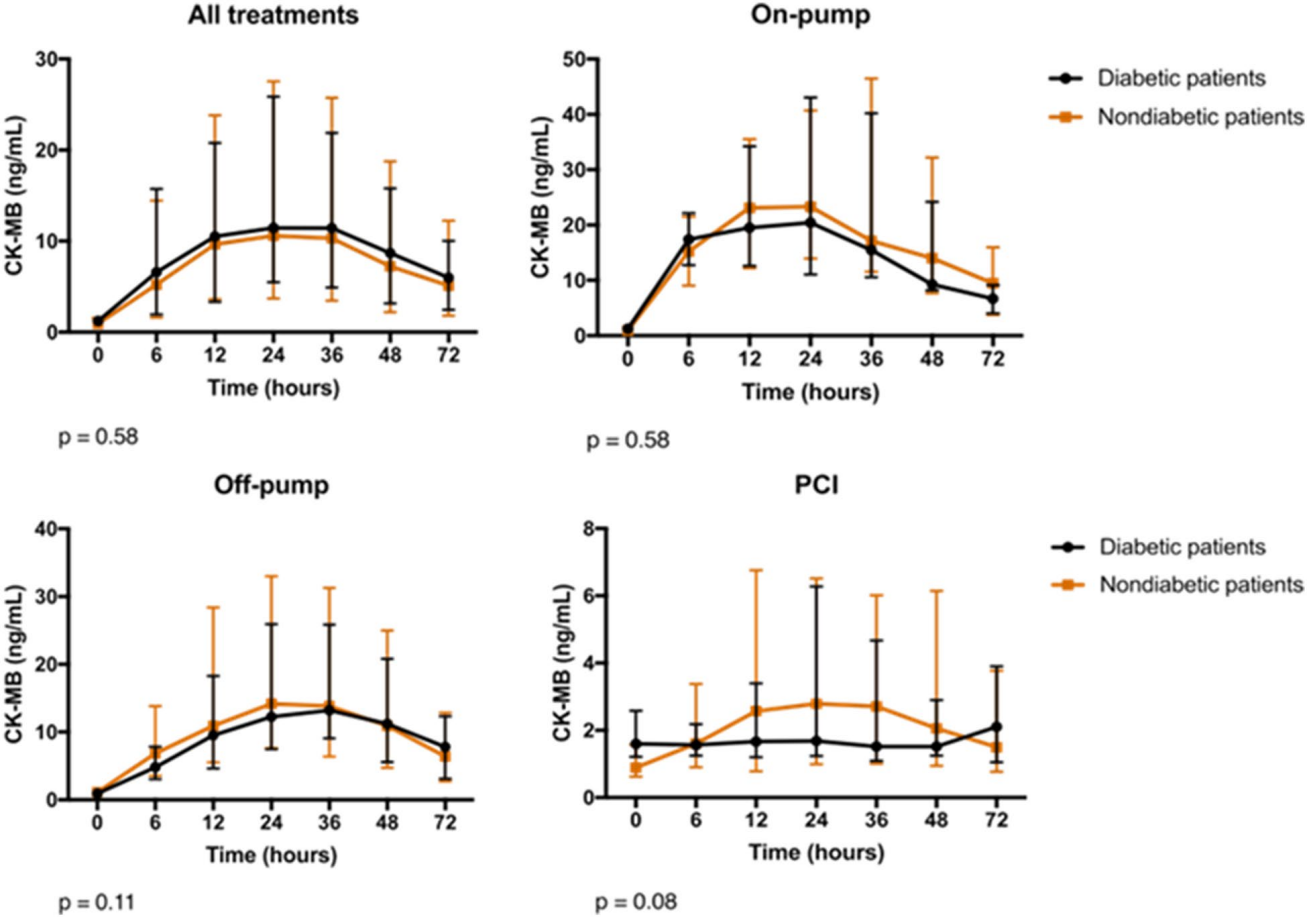
CK-MB results at baseline and after 6, 12, 24, 36, 48, and 72 h after coronary interventions in diabetic and nondiabetic patients. Data are presented as medians and interquartile ranges. *P* values are provided by the comparison of both groups regarding all time points

We also compared CK-MB values at baseline and during each time point (6, 12, 24, 36, 48, and 72 h) between diabetic and nondiabetic patients and separately for each cardiac procedure (Fig. 2).

### CMR results

**Fig. 3:**
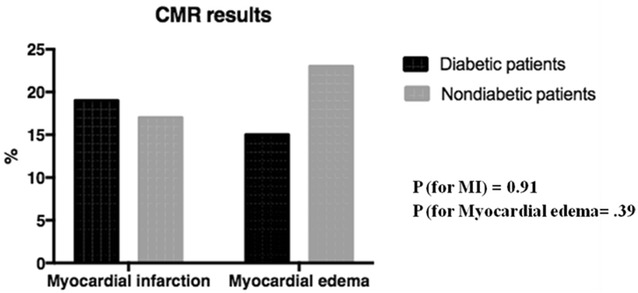
Myocardial fibrosis and edema after coronary interventions in diabetic and nondiabetic patients, according to CMR analyses

New LGE on CMR indicating new myocardial fibrosis after interventions was present in 18.9% and 17.3% (*P* = 0.91), and myocardial edema in 15.5% and 22.9% (*P* = 0.39) in diabetic and nondiabetic patients, respectively (Fig. 3).

### Analyses of correlation

Peak CK-MB and peak troponin were plotted against fasting glycemic and A1c levels to look for any correlations among these variables. Glycemic levels and peak troponin had a very weak negative correlation (r = − 0.08, 95% CI − 0.22 to 0.07, *P* = 0.28). Glycemic levels and peak CK-MB also had a very weak correlation (r = 0.01, 95% CI − 0.13 to 0.16, *P* = 0.85).

The analysis of correlation of A1c levels from diabetic patients also showed a very weak negative correlation with peak troponin and CK-MB levels (r = − 0.18, 95% CI − 0.38 to 0.03, *P* = 0.08 for troponin; r = − 0.14, 95% CI − 0.34 to 0.08, *P* = 0.20 for CK-MB).

### Periprocedural myocardial infarction on CMR according to tertiles of glycemia and A1c levels

**Fig. 4:**
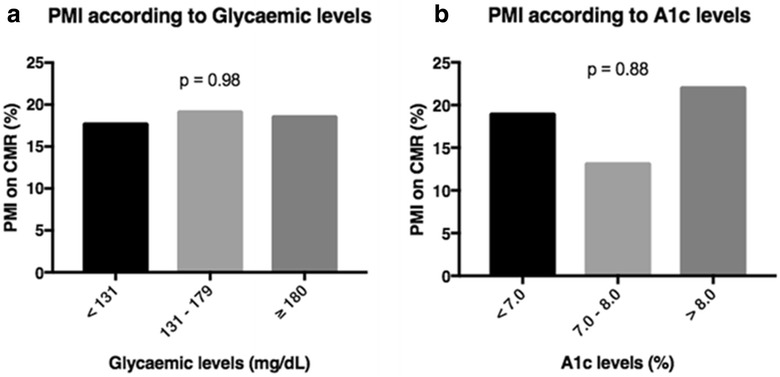
**a**, **b** Proportion of periprocedural myocardial infarction on cardiac magnetic resonance according to glycemic (**a**) and A1c (**b**) levels at baseline

Of 202 patients, 200 had their baseline glycemia measured. These patients were grouped into tertiles of glycemia (< 131, n = 134; 131–179, n = 31; ≥ 180, n = 32). No difference was found regarding PMI on CMR (17.9% versus 19.3% versus 18.7%, *P* = 0.98). These results are shown in Fig. 4a.

From 90 diabetic patients, 89 had their baseline A1c levels measured. These patients were grouped into tertiles of A1c (< 7.0, n = 47; 7.0–8.0, n = 15; > 8.0, n = 27). No difference was also found regarding PMI on CMR (19.1% versus 13.3% versus 22.2%, *P* = 0.88). These results are shown in Fig. 4b.

## Discussion

The analysis of the data from this study shows that patients with diabetes have similar myocardial sensitivity to that of nondiabetic patients after revascularization interventions. The evidence of similar release of both cardiac biomarkers (troponin and CK-MB) after three distinct revascularization strategies, PCI, on-pump, or off-pump CABG, was confirmed by the findings of a high-sensitive and specific imaging technique to detect cardiac ischemic damage, the cardiac magnetic resonance, by the analysis of necrosis as well as myocardial edema.

Although the release of markers of myocardial necrosis after interventions are multifactorial and do not necessarily represent ischemic insults, the findings of myocardial necrosis and edema at CMR are mostly related to ischemic events during cardiac procedures and can be considered as strong evidence to confirm these findings in our sample. In fact, the strength of the present study lies in the assessment of multiple time points of both cardiac biomarkers collected in a standardized fashion, with a minimum loss of data, and by bringing the relevant information of CMR results, for both myocardial necrosis and edema. Thus, the analysis of the release of these biomarkers and the findings of cardiac resonance shows similar myocardial sensitivity between diabetic and nondiabetic patients in this sample.

An interesting study from Verdoia et al. evaluated the effect of diabetes in PMI and myonecrosis after PCI treatment [18]. Using the release of CK-MB and troponin to define PMI and myonecrosis, the authors found that diabetes is not associated with increased risk of cardiac damage after PCI therapy. Thus, their findings agree with the results from the present study. However, adding to the information from that study, the present one also assessed the release of biomarkers after on-pump and off-pump bypass surgery and the evaluation of cardiac magnetic resonance.

Other studies have assessed the release of cardiac biomarkers in the setting of acute coronary syndromes and also evaluated mortality rates in diabetic patients [13, 19–22]. Interestingly, although the majority of them have found higher mortality rates in the diabetic population, the release of cardiac biomarkers was similar to that in the nondiabetic population, the same finding seen in the present study after coronary interventions. Thus, these studies show that myocardial sensitivity is probably not influenced by diabetes status, and other factors might be associated with the higher morbidity and mortality rates observed in the diabetic population. Potential reasons for this observation are speculative and still under debate, but might include greater extent of coronary artery disease [23], systolic dysfunction [24], diastolic dysfunction [8], and a theoretically pro-thrombotic state that might be associated with diabetes [25]. Although some argue that myocardial intrinsic properties such as ischemic preconditioning might be altered by diabetes [26], our group has recently shown similar findings between diabetic and nondiabetic patients who underwent sequential exercise tests, as part of a preconditioning protocol [27].

In agreement with the findings of this study, Alegria et al. [28] have also evaluated mortality rates at 6 months and infarct size by myocardial scintigraphy in patients who suffered an acute myocardial infarction treated by thrombolytic therapy. They have shown higher mortality rates associated with diabetes that persisted after adjustment for confounding factors. However, this higher mortality could not be totally explained by very modest differences in myocardial infarct size and reduced ejection fraction in diabetic patients. Moreover, the authors could not completely rule out that these very small differences could not still be due to the remaining influence of confounders.

Furthermore, a recent study by Grodzinsky et al. [29] has also assessed angina symptoms over 1 year after PCI treatment. In this study, angina prevalence and severity were similar between patients with and without diabetes. These findings also confirm the findings of the present study: ischemia assessed by the presence of angina and its effects in the myocardium of patients after coronary interventions occur independently of diabetes status.

Interestingly, the analysis of correlation between glycemic and A1c levels and peak troponin and CK-MB did not show any correlation among these variables, although graphically it seems that the higher levels of troponin and CK-MB were found in the lower levels of glycemia. These findings might probably be because the higher peak levels of cardiac markers occurred within the most frequent observations of glycemic levels. In addition, the correlation coefficients do not support any further conclusions. These results are also confirmed by the analysis of cardiac damage assessed by magnetic resonance that did not show any association of PMI with glycemic and A1c level groups at baseline.

The results from this study deserve some consideration. First, it is possible that the small sample size could limit the study power to detect differences between the two populations. On the other hand, the multiple measurements of cardiac biomarkers in addition to the findings of cardiac resonance make this possibility less reasonable. Second, the well-controlled glycemic status, noted by the well-controlled levels of fasting glycemia and A1c in the diabetic population might have influenced study findings. Different findings could be observed in a diabetic population with poor controlled glycemic levels.

## Conclusions

In this study, type 2 diabetes mellitus did not add risk to the development of myocardial injury after percutaneous coronary intervention or bypass surgery in patients with multivessel coronary artery disease.

## Abbreviations

A1c: glycated hemoglobin
CABG: coronary artery bypass graft
CAD: coronary artery disease
CMR: cardiac magnetic resonance
IQR: interquartile range
LGE: late gadolinium enhancement
MASS V: medicine, angioplasty, or surgery study V
PCI: percutaneous coronary intervention
PMI: periprocedural myocardial infarction
TnI: troponin I
